# Evolution of Spatial and Temporal *cis-*Regulatory Divergence in Sticklebacks

**DOI:** 10.1093/molbev/msad034

**Published:** 2023-02-20

**Authors:** Katya L Mack, Tyler A Square, Bin Zhao, Craig T Miller, Hunter B Fraser

**Affiliations:** Department of Biology, Stanford University, Stanford, CA; Department of Molecular and Cell Biology, University of California, Berkeley, CA; Department of Biology, Stanford University, Stanford, CA; Department of Molecular and Cell Biology, University of California, Berkeley, CA; Department of Biology, Stanford University, Stanford, CA

**Keywords:** *cis*-regulatory evolution, adaptation, gene expression, sticklebacks

## Abstract

*Cis-*regulatory changes are thought to play a major role in adaptation. Threespine sticklebacks have repeatedly colonized freshwater habitats in the Northern Hemisphere, where they have evolved a suite of phenotypes that distinguish them from marine populations, including changes in physiology, behavior, and morphology. To understand the role of gene regulatory evolution in adaptive divergence, here we investigate *cis-*regulatory changes in gene expression between marine and freshwater ecotypes through allele-specific expression (ASE) in F1 hybrids. Surveying seven ecologically relevant tissues, including three sampled across two developmental stages, we identified *cis-*regulatory divergence affecting a third of genes, nearly half of which were tissue-specific. Next, we compared allele-specific expression in dental tissues at two timepoints to characterize *cis*-regulatory changes during development between marine and freshwater fish. Applying a genome-wide test for selection on *cis*-regulatory changes, we find evidence for lineage-specific selection on several processes between ecotypes, including the Wnt signaling pathway in dental tissues. Finally, we show that genes with ASE, particularly those that are tissue-specific, are strongly enriched in genomic regions of repeated marine-freshwater divergence, supporting an important role for these *cis*-regulatory differences in parallel adaptive evolution of sticklebacks to freshwater habitats. Altogether, our results provide insight into the *cis-*regulatory landscape of divergence between stickleback ecotypes across tissues and during development, and support a fundamental role for tissue-specific *cis*-regulatory changes in rapid adaptation to new environments.

## Introduction

Understanding how organisms adapt to new environments is a major goal in evolutionary biology. Central to this goal is understanding what genetic changes underlie adaptive traits. Threespine sticklebacks (*Gasterosteus aculeatus*) are a powerful model for studying the genetic basis of adaptation (Reid et al. 2021). After the end of the last ice age, marine sticklebacks colonized thousands of freshwater habitats in the Northern Hemisphere (Bell and Foster 1994). In these freshwater environments, populations have rapidly evolved many traits that distinguish them from the ancestral marine form. Although adaptation to many lakes or streams is independent, several traits have evolved repeatedly across multiple freshwater systems either through parallel, convergent, or distinct genetic changes (e.g., changes in body shape, skeletal armor, dentition, behavior, and pigmentation) (Bell and Foster 1994; Walker and Bell 2000; Colosimo et al. 2005; Miller et al. 2007; Cleves et al. 2014). The repeated evolution of similar phenotypes in freshwater systems is strong evidence that these traits reflect local adaptation and provide a powerful platform for studying the genetic architecture of adaptive phenotypic evolution (Jones et al. 2012; O’Brown et al. 2015; Roberts Kingman, Vyas, et al. 2021).

Mutations in *cis-*regulatory elements can change how nearby genes are regulated. Such mutations are thought to be an important substrate for adaptive evolution (Prud’homme et al. 2007; Stern and Orgogozo 2009; Signor and Nuzhdin 2018). In contrast to protein-coding changes, *cis-*regulatory mutations can alter the expression of gene targets in tissue- or temporally-specific ways. As a consequence, *cis-*regulatory changes may be less constrained by the deleterious side effects of negative pleiotropy, making this class of mutations important targets for natural selection (Prud’homme et al. 2007; Stern and Orgogozo 2009). *Cis*-regulation has been shown to be the major driver of local environmental adaptation in recent human evolution (Fraser 2013), and likewise, plays a central role in the local adaptation of sticklebacks to freshwater environments. Genome scans have found that genomic regions associated with a recurrent divergence between ecotypes are predominantly intergenic, suggesting that parallel divergence may often involve the reuse of pre-existing gene regulatory variation (Jones et al. 2012). *Cis-*regulatory mutations have been implicated in specific morphological differences between marine and freshwater forms, including the loss of pelvic spines (Chan et al. 2010), bony armor plates (O’Brown et al. 2015), changes in pigmentation (Miller et al. 2007), and increased pharyngeal tooth number (Cleves et al. 2014, p. 6; Stepaniak et al. 2021). Although these lines of evidence suggest an important role for gene regulatory evolution in stickleback adaptation, the global *cis-*regulatory landscape of marine-freshwater divergence remains poorly understood. Exploration of *cis-*regulatory changes between ecotypes has largely been limited to assaying individual gene targets in a small number of tissues (e.g., Cleves et al. 2014, p. 6; O’Brown et al. 2015; Howes et al. 2017; Roberts Kingman, Lee, et al. 2021). Transcriptome-wide *cis-*regulatory divergence between marine and freshwater fish has been characterized in two tissues so far: the gills (Verta and Jones 2019) and ventral pharyngeal tooth plates (Hart et al. 2018). These two tissues showed highly divergent regulatory landscapes (Verta and Jones 2019), with *cis-*regulatory changes playing a predominant role in expression divergence in the gills but not in the ventral pharyngeal tooth plates. This contrast suggests that stickleback tissues may vary not only in the specific genes with regulatory divergence, but also in the genome-wide prevalence of *cis* versus *trans*-acting changes and the respective contribution of these changes to parallel adaptive evolution (Verta and Jones 2019).

Here, we survey global *cis-*regulatory divergence between marine and freshwater sticklebacks in seven tissues to understand the role of gene expression evolution in adaptive divergence. To characterize *cis*-regulatory changes between ecotypes, we crossed marine and freshwater fish to generate F1 hybrids. As F1 hybrids carry both a marine and freshwater copy of each chromosome, alleles from both parents are present in the same cellular environment (e.g., subject to the same *trans*-acting factors). Expression differences between the two parental alleles (i.e., allele-specific expression) can, therefore, only result from *cis-*regulatory changes (Cowles et al. 2002; Wittkopp et al. 2004). We use this approach to examine a collection of tissues important for behavioral, physiological, feeding, and morphology differences between the marine and freshwater forms (i.e., brain, liver, eyes, flank skin, dorsal and ventral pharyngeal tooth plates, and mandible). Brain and liver were examined because of the potential role of these tissues in behavioral and physiological differences between ecotypes (Wark et al. 2011; Di Poi et al. 2016; Reid et al. 2021). Eyes have also been shown to differ in morphology and spectral sensitivity (Rennison et al. 2016). Flank skin was examined as the location where lateral armor plates develop in adulthood (Bell and Foster 1994; O’Brown et al. 2015). As morphological changes include especially dramatic changes to the craniofacial skeleton and dentition (Cleves et al. 2014; Miller et al. 2014), likely reflecting adaptations to different diets in freshwater, we also examine two developmental timepoints in dental tissues to characterize *cis*-regulatory modifications during development. We use these data to dissect the landscape of *cis*-regulatory divergence and then ask whether these changes are associated with the genomic signals of selection. Overall, our results highlight the importance of the tissue- and developmental stage-specific *cis*-regulatory changes in marine-freshwater divergence and the importance of this *cis-*regulatory variation to local adaptation.

## Results and Discussion

### Extensive Allele-Specific Expression Across Tissues in Freshwater-Marine Hybrids

**Fig. 1.**
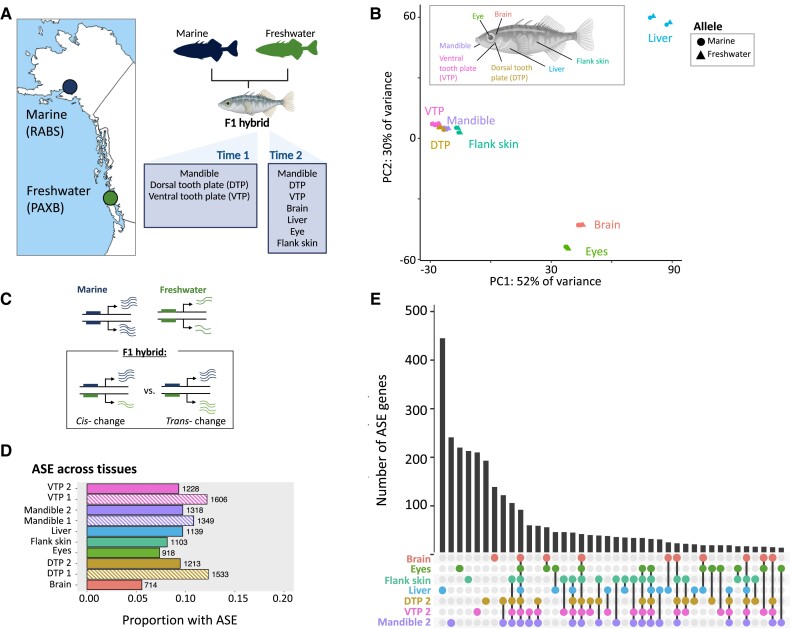
The *cis*-regulatory landscape of divergence between marine and freshwater sticklebacks. (*A*) Marine (RABS) and freshwater (PAXB) sticklebacks were crossed to produce F1 hybrids. Tissues were collected for RNAseq across two developmental timepoints from full siblings. (*B*) Principal component analysis of allelic counts from marine-freshwater F1 hybrids. Allelic reads cluster by the tissue of origin on PC1 and PC2. (*C*) A schematic of how regulatory divergence can be dissected with F1 hybrids. Here, a gene is upregulated in marine fish (wavy lines). In an F1 hybrid, differential expression between the freshwater and marine allele (e.g., allele-specific expression, ASE) indicates a *cis*-regulatory change. In contrast, equal expression of the two alleles indicates a *trans-* only change. (*D*) Numbers and proportions of genes with ASE across tissues. Striped bars indicate tissues from the early timepoint. (*E*) UpSet plot showing distinct intersections of genes with ASE across tissues from the late timepoint. A single dot in a column indicates ASE specific to one tissue, whereas multiple dots connected by lines indicate ASE shared across multiple tissues.

To investigate *cis-*regulatory divergence between marine and freshwater individuals, we analyzed allele-specific expression in F1 hybrids between marine and freshwater fish (freshwater Paxton lake benthic [PAXB] × marine Rabbit Slough [RABS]) (fig. 1*A*). Seven tissues were collected from F1 hybrids at the young adult stage (∼35 millimeters [mm] standard length [SL]) (brain, eyes, liver, flank skin, ventral pharyngeal tooth plate [VTP], dorsal pharyngeal tooth plate [DTP], and mandible) (figs. 1*A* and *B*). Additionally, three dental tissues (mandible, VTP, and DTP) were also collected from full-siblings at an earlier juvenile stage (15–20 mm SL) for the temporal comparison of dental development (hereafter, “early” vs. “late” developmental timepoint). We sequenced mRNA from each tissue for two biological replicates, obtaining a median of 66.7 million reads per sample (supplementary tables S1 and S2, Supplementary Material online). To phase heterozygous sites in F1s, we also performed the whole-genome sequencing of the freshwater parent (PAXB) to an average coverage of ∼30 × (supplementary fig. S1, Supplementary Material online).

Principal component (PC) analysis of gene-wise mRNA abundance and allele-specific expression (ASE) revealed that tissue type is the primary driver of variation (fig. 1*B* and supplementary fig. S2, Supplementary Material online). Allele-specific expression values clustered largely by the tissue of origin on PC1 and PC2 (PC1: 52% of the variation, PC2: 30% of variance). Dental tissues formed their own cluster to the exclusion of other tissues, as did the eyes and the brain. Flank skin, where bony lateral plates develop, also formed a group with dental tissues on PC1 (fig. 1*B*). PC analysis of the allele-specific expression of dental tissue timepoints also separated samples based on the developmental stage (early vs. late) on PC1 or PC2 (supplementary fig. S3, Supplementary Material online).

Extensive ASE was found across tissues (fig. 1*C* and *D*). Nearly 33% of genes (4,411) were found to have significant ASE in at least one tissue or tissue-timepoint (DESeq2 Wald-test, FDR < 0.05, see supplementary table S3, Supplementary Material online, fig. 1*D*; 13,551 genes tested). In each tissue, these ASE genes accounted for approximately 5–12% of the genes surveyed. Dental tissues had the greatest number of ASE genes overall, particularly at the earlier developmental timepoint (fig. 1*D*). The lowest number of ASE genes was identified in the brain (714 genes, 5.6%). The number of ASE genes identified in a tissue was not related to differences in read depth between tissues (supplementary fig. S4, Supplementary Material online).

Comparing the overlap of genes with ASE between tissues, we found that the largest distinct groups are tissue-specific rather than shared, indicating largely tissue-specific *cis-*regulatory divergence between marine and freshwater fish (fig. 1*E*). Across the seven tissues sampled at the late timepoint (SL ∼35 mm), 1,660 genes showed ASE in only one tissue (48% of genes with ASE overall). In particular, the liver was found to have the greatest number of unique ASE genes (366 genes, 32% of genes with ASE in the liver). Comparisons between tissues also revealed many genes with evidence for shared ASE (fig. 1*E*). In particular, we found a high overlap between eyes and brain (29% shared), between dental tissues (36–44%), and between dental tissues and flank skin (32–36%) (supplementary table S4, Supplementary Material online). In contrast, few genes (∼2%) showed ASE across all tissues (78 genes across all tissues, 92 genes at the late developmental timepoint). For genes with ASE in multiple tissues, directionality was typically maintained, with only 236 genes showing a change in which the parental allele was upregulated between tissues.

### Widespread Heterogeneity in Allele-Specific Expression Across Tissues in Marine-Freshwater Hybrids

Comparisons of ASE across tissues revealed abundant *cis-*regulatory divergence between marine and freshwater fish. To investigate variation in allele-specific expression between tissues in marine-freshwater hybrids, we employed a Bayesian approach to partition genes in each tissue into one of three states—no ASE, moderate ASE, and strong ASE—based on the numbers of reads supporting the marine and freshwater allele (Pirinen et al. 2015). Tissues are further classified as showing ASE heterogeneity if the strength of ASE varies across tissues (e.g., ASE is present in some tissues but absent in others or varies in magnitude between different tissues). Finally, we consider a substate of ASE heterogeneity to be tissue-specificity, where the ASE state (i.e., moderate, strong ASE, or no ASE) is observed in only one tissue despite expression of the gene across multiple tissues. Consequently, tissue-specificity describes cases where the ASE state is unique to a single tissue.

**Fig. 2.**
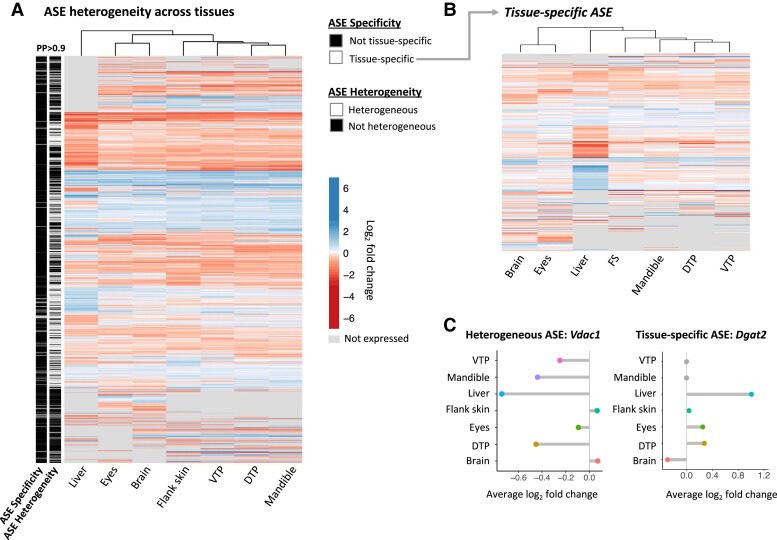
Heterogeneity of allele-specific expression across tissues. (*A*) A heatmap of genes with evidence of allele-specific expression in late development. The left bars indicate genes where ASE varies across tissues (in presence or magnitude, “ASE Heterogeneity”) and a substate of ASE heterogeneity where ASE patterns are specific to one tissue (tissue-specific ASE, “ASE Specificity”) at a posterior probability of >0.9. Genes are colored by average log_2_ fold change in each tissue, the difference in expression between the marine and freshwater allele in the F1 hybrid. Gray panels indicate that the gene is not expressed in a given tissue. (*B*) Heatmap of genes with evidence for tissue-specific ASE. (*C*) Examples of genes with heterogeneous ASE (*Vdac1,* left) and tissue-specific ASE (*Dgat2*, right). ASE was observed for voltage-dependent anion channel *Vdac1* in some tissues (e.g., liver, DTP2, mandible) but not others, where triglyceride synthesis gene *Dgat2* only shows evidence for ASE in the liver.

We found that heterogeneity in ASE between tissues was common. Comparing across the seven different tissues collected at our second timepoint, we found that 44% of genes with ASE are classified as having heterogeneous ASE at a posterior probability (PP) > 0.9 (at PP > 0.95, 38%) (fig. 2*A*; Full list in supplementary file S1, Supplementary Material online). Nearly all the genes with ASE heterogeneity (99%) did not show ASE in at least one of the tissues surveyed, with the remaining 1% showing evidence for ASE of varying magnitudes across tissues. Evidence of tissue-specificity was also found for 448 genes (PP > 0.9, supplementary file S1, Supplementary Material online) (fig. 2*B*). Liver harbored the greatest number of genes with tissue-specific ASE (141 genes), followed by the eyes (66 genes). Repeating this analysis to incorporate dental tissues from both the early and late timepoints, we also identified 73 genes with developmental- and tissue-specific ASE in tissues from the early developmental stage (supplementary file S1, Supplementary Material online). Overall, allele-specific expression across tissues was found to be highly heterogeneous, likely reflecting tissue-specific *cis-*regulatory differences between marine and freshwater individuals.

Several genes with tissue-specific ASE were of interest for their reported tissue-specific functions in other systems (supplementary file S1, Supplementary Material online). For example, although *Dgat2* is expressed in five tissues at the second timepoint, its ASE is confined to liver (fig. 2*C*). *Dgat2* is involved in triglyceride synthesis and plays an important role in energy metabolism; in mammals and zebrafish, gene mutants are associated with fatty liver (Choi et al. 2007; O’Hare et al. 2017, p. 6). In dental tissues, genes with tissue-specific expression include several genes involved in tooth and bone formation (e.g., *Spp1*, *Dlx1a, Odam, Sox2, Epha3*, *Ssuh2rs1, Tgfbr2b, Stc2a*) (supplementary file S1, Supplementary Material online). *Stc2a*, which was found to have tissue-specific ASE in the early mandible, was also recently shown to underlie changes in pelvic spine length between stickleback populations (Roberts Kingman, Lee, et al. 2021).

### Temporal Differences in Allele-Specific Expression During Dental Development

**Fig. 3.**
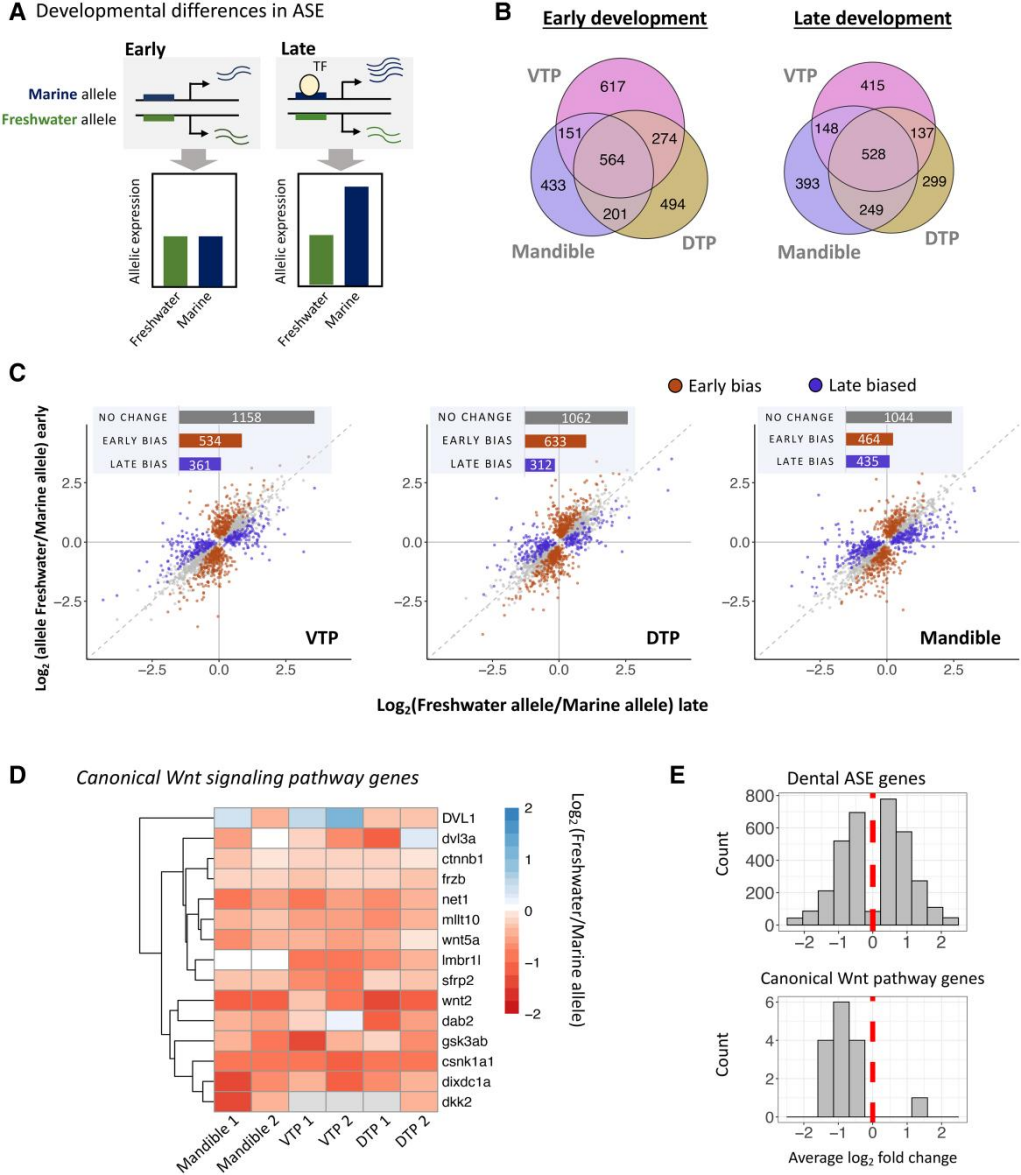
Developmental allele-specific expression in dental tissues. (*A*) A schematic of differential ASE during development. In this example, sequence divergence between marine and freshwater sticklebacks at a *cis*-regulatory region results in allele-specific expression only in the presence of a context-specific transcription factor (“TF”, circle) expressed during late development. This results in differential allele-specific between developmental stages, shown in the bar plots. (*B*) Venn diagrams of ASE in dental tissues at the early (left) and late (right) developmental timepoints. (*C*) Temporal differences in ASE during development in three dental tissues (left to right: ventral tooth plate, dorsal tooth plate, mandible). Genes with differential allele-specific between timepoints are colored based on the magnitude of ASE in timepoint 1 versus 2. Genes with greater differences in allelic expression in early development are shown in purple (“early bias”), genes with greater expression differences at the late timepoint are shown in terracotta (“late bias”). Gray points/bar (“no change”) indicate genes without evidence for significant differential allele-specific expression between timepoints. (*D* and *E*) Genes involved in Canonical Wnt signaling show evidence of polygenic *cis-*regulatory evolution in dental tissues. Here, we show genes from two Wnt signaling GO terms with biased directionality (Canonical Wnt signaling [GO:0060070]; Negative regulation of canonical Wnt signaling [GO:0090090]) (supplementary table S7, Supplementary Material online). In (*D*), the heatmap shows log_2_ fold changes for genes associated with canonical Wnt signaling and ASE in at least one dental tissue. In (*E*), histograms of average ASE gene log_2_ fold changes from all dental genes (top) and the canonical Wnt signaling gene set (bottom). For each gene, log_2_ fold changes are averaged across any dental tissues in which ASE is identified.

Marine and freshwater sticklebacks show a number of phenotypic differences associated with feeding morphology (e.g., larger jaws, more teeth), likely reflecting adaptations to larger prey found in the benthic zone of lakes (Lavin and McPhail 1986; Bell and Foster 1994). Divergence in tooth number arises during late development, providing an opportunity to study *cis-*regulatory divergence in the context of developmental evolution (Cleves et al. 2014; Ellis et al. 2015; Hart et al. 2018). To investigate *cis-*regulatory divergence during dental development, we examined ASE in three dental tissues at two developmental timepoints (fig. 3*A*).

Developmental stage was a major component of variation in allele-specific expression. Principal component analysis of marine-freshwater allelic log_2_ fold changes clustered tissues by timepoint, with late and early dental tissues forming separate clusters on PC2 (22% of the variance, supplementary fig. S5, Supplementary Material online). PC analysis of allele-specific counts from individual tissues also clustered tissues based on the developmental timepoint on either PC1 (mandible and DTP, 50% and 48% of variance, respectively) or PC2 (VTP, 33% of variance) (supplementary fig. S3, Supplementary Material online).

In contrast to our comparison of more diverse tissues, dental ASE is often shared across tissues or developmental stages (fig. 3*B*, supplementary fig. S6, Supplementary Material online). Nearly 10% of genes with ASE in dental tissues (330/3,471 genes) showed ASE in all three dental tissues and at both developmental timepoints. Overall, a greater proportion of genes with ASE were shared across dental tissues in late development compared to early development: 19% of ASE genes (564 genes) were shared across all three tissues in the early stage versus 26% (528 genes) in the late stage (Chi-square test, *P* = 0.002). Examining the stickleback orthologs of genes implicated in mammalian tooth development collected from the Bite-It and ToothCode databases (hereafter, referred to as “BiteCode” genes (Hart et al. 2018)), we found that these genes were enriched for ASE (Fisher's exact test, *P* = 0.0046) (supplementary fig. S5*B*, Supplementary Material online). BiteCode enrichment is consistent with the conservation of regulatory networks regulating dental development in mammals and fish (Stock 2007; Fraser et al. 2009).

Next, we characterized developmental differences in *cis-*regulatory divergence by comparing ASE between timepoints. Across developmental stages, divergent ASE can reflect the activity of temporally-specific genes controlled by divergent *cis-*regulatory elements between marine and freshwater fish (fig. 3*A*). Comparing the ratio of marine to freshwater allelic counts between early and late development in hybrids, we observed a widespread differential allele-specific expression between the two developmental stages for each tissue, accounting for 37–43% of genes with ASE at either timepoint (fig. 3*C*) (Fisher's exact tests, FDR < 0.05; see Methods). The majority of differential ASE reflected ASE that was timepoint specific, meaning ASE was only observed at one developmental stage. However, roughly a quarter of differential ASE in each tissue was due to changes in the magnitude of ASE between timepoints.

More genes with differential ASE are found to have a larger *cis*-effect at the early stage than the late stage (i.e., | log2 fold change in early | > | log2 fold change in late |); fig. 3*C*), consistent with the greater proportion of genes with ASE at the early timepoint overall (fig. 1*D*). This result was surprising, as greater phenotypic divergence is observed between marine and freshwater fish in the pharyngeal tooth plates in late development (Ellis et al. 2015). Developmental differences were also typically tissue-specific: 67% of genes with developmental stage-bias ASE were unique to one tissue. Thus, *cis*-regulatory differences between marine and freshwater individuals are often specific to both tissue and tissue-developmental stage.

### Polygenic Selection on *cis*-Regulatory Divergence Between Marine and Freshwater Sticklebacks

*Cis*-regulatory changes between marine and freshwater sticklebacks are potentially interesting for their roles in local adaptation (Jones et al. 2012). However, the majority of *cis-*regulatory changes are expected to be neutral. To test for selection on *cis*-regulatory changes between marine and freshwater fish, we employed a gene-set approach based on the sign test framework (Bullard et al. 2010; Fraser et al. 2011). Under neutrality, quantitative trait loci (QTLs) for any given trait are expected to be unbiased with respect to their directionality, assuming that these QTLs are independent (i.e., caused by different genetic variants) (Orr 1998). In a marine/freshwater genetic cross, each allele would be expected to be equally likely to increase the trait value if that trait is not under lineage-specific selection. Similarly, if a gene set associated with a biological function shows a significant directional bias in ASE (with more *cis-*changes acting in the same direction than expected), this suggests lineage-specific selection on the *cis*-regulation of this gene set (Bullard et al. 2010; Fraser et al. 2011). Applying the sign test to GO gene sets in individual tissues and to the combined dental tissue set, we identified multiple gene sets with evidence for biased directionality (full list in supplementary tables S5 and S6, Supplementary Material online).

In the combined dental tissue set, we found the strongest biased directionality for the GO term “canonical Wnt signaling pathway” (Permutation based *P*-value = 0.0078). Wnt signaling plays a critical and evolutionarily conserved role in tooth and bone development (Liu et al. 2008; Square et al. 2021) and genes in this pathway with ASE have been directly implicated in regulating dental development in other species (e.g., *Wnt5a*, *Sfrp2*, *Ctnnb1*, *Net1*) (supplementary table S7, Supplementary Material online). Although the GO annotation term includes both positive and negative regulators of Wnt signaling, the pathway “negative regulation of canonical Wnt signaling” is also nominally significant for the biased downregulation of freshwater alleles (7/7 genes, Fisher's exact test, *P* = 0.0048), suggestive of the biased Wnt inhibition in marine fish (figs. 3*D* and *E*). Only three positive regulators of canonical Wnt signaling had ASE in dental tissues, precluding a separate statistical test of their directionality. As the disruption or inhibition of canonical Wnt signaling results in arrested/aberrant tooth formation, selection on this pathway could potentially reflect selection for increased tooth number or related changes in feeding morphology in freshwater fish. Consistent with this, genes in the Wnt signaling pathway were previously shown to be upregulated in the VTP in PAXB freshwater compared to marine fish (Hart et al. 2018).

The second most significant gene set under lineage-specific selection was the GO term “embryonic viscerocranium morphogenesis” (*P* = 0.0093). These genes are involved in the generation and organization of the facial skeleton, and also include ASE genes directly implicated in tooth and jaw formation (supplementary table S8, Supplementary Material online). For instance, *Dlx3b* and *Dlx1a*, genes encoding members of the Dlx family of homeodomain transcription factors (Kraus and Lufkin 2006), are involved in tooth and jaw patterning in mammals and fish (Jeong et al. 2008; Pang et al. 2020). Thus, the biased directionality of this process category may reflect selection for morphological changes to freshwater fish in the facial region related to feeding morphology. However, it is difficult to make a confident prediction of what specific trait(s) these concerted expression changes may influence, since they could impact many aspects of craniofacial morphology. For this reason, the sign test is generally expected to be most powerful when applied to gene sets with shared directionality of effects on a selected trait.

We also found biased directionality for gene sets in individual tissues (supplementary table S5, Supplementary Material online). For instance, the GO category term “methyltransferase activity” (*P* = 0.0018, 10/10 terms) showed biased upregulation of marine alleles in the eye and “endoplasmic reticulum” (*P* = 0.0039, 38/50) showed biased upregulation of marine alleles in the flank skin. Since these gene sets are not yet associated with specific phenotypes, it is unclear which traits may have been impacted by their lineage-specific selection.

### Overlap Between Signatures of Selection and Genes With *cis*-Regulatory Divergence

If *cis-*regulatory changes underlie adaptive divergence between freshwater and marine forms, we may expect genes with ASE to fall within or near regions with signatures of selection. To test this hypothesis, we utilized a recent whole-genome analysis of differentiation between marine and freshwater populations from the northeast Pacific basin (Roberts Kingman, Vyas, et al. 2021) (the source of the freshwater PAXB population studied here), where genomic regions of repeated marine-freshwater divergence were identified through marine-freshwater cluster separation scores (CSS). A CSS score quantifies average marine-freshwater genetic distance after subtracting the genetic distance found within each ecotype for a genomic window (Jones et al. 2012; Roberts Kingman, Vyas, et al. 2021).

**Fig. 4.**
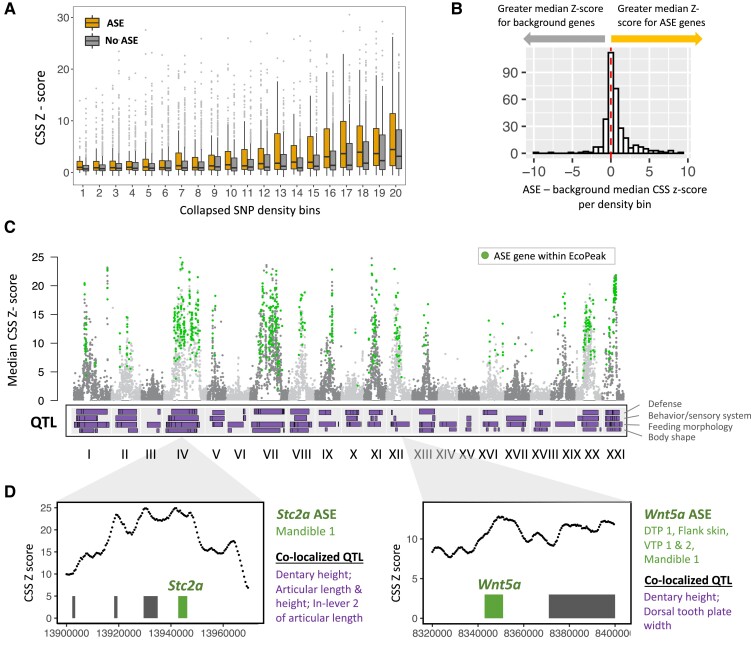
ASE genes are associated with regions of repeated marine-freshwater divergence. (*A*) Average marine-freshwater cluster separation score (CSS) Z-scores for ASE genes and background genes binned by SNP density. Here, genes are separated into 20 density bins for visualization, with higher numbers corresponding to greater SNP density. (*B*) ASE genes have higher average CSS Z-scores than background genes. The histogram shows median Z-scores for ASE genes minus background genes for each SNP density bin. More density bins show positive values, indicating higher average Z-scores for ASE genes overall (Permutation *P* < 0.0001). (*C*) Manhattan plot of median gene CSS Z-score versus chromosome position. Highlighted in green are genes with ASE that overlap significant regions of recurrent marine-freshwater divergence in the northeast Pacific basin (“EcoPeaks”). Below, we show locations of quantitative trait loci (QTLs) identified in previous genetic crosses between PAXB and marine fish. QTLs are divided into three broad categories (from top to bottom: defense, behavior, and sensory system, feeding morphology, and body shape). (*D*) Two candidate ASE genes within regions of marine-freshwater divergence. ASE was observed for *Wnt5a* and *Stc2a* in one or more dental tissues and these genes co-localize with QTL related to feeding morphology. Bars in the panel indicate genes within these regions, with candidate genes *Wnt5a* and *Stc2a* highlighted in green. Tissue(*s*) in which ASE was identified (green) and relevant overlapping QTL (purple) are listed to the right of each gene panel.

We asked whether genes with ASE colocalized with genomic regions with greater evidence for marine-freshwater divergence (i.e., greater CSS Z-scores). As power to detect ASE is related to the number of variant sites, we compared median CSS Z-scores between ASE and background genes with similar SNP densities (see Methods, supplementary fig. S7, Supplementary Material online). Genes with ASE are associated with greater Z-scores per SNP density bin (figs. 4*A* and *B*; Permutation *P* < 0.0001), indicating an enrichment of ASE genes in genomic regions with greater evidence for marine-freshwater divergence. This pattern is consistent with the hypothesis that repeated marine-freshwater divergence may often involve changes in gene regulation (Jones et al. 2012; Verta and Jones 2019).

Regions with significant CSS scores (EcoPeaks) overlapped 611 ASE genes (13.8% of ASE genes overall; 1.9-fold enrichment, Permutation test *P* < 0.001) (fig. 4*C*, supplementary table S9, Supplementary Material online). Genes with evidence for ASE heterogeneity between tissues were enriched within EcoPeaks compared to all genes with evidence for ASE (Fisher's exact test, *P* = 0.01), as were genes with evidence for tissue-specific ASE (Fisher's exact test, *P* = 0.018).

Marine-freshwater EcoPeaks are clustered throughout the genome, which is thought to reflect selection on linked “supergene” complexes affecting multiple traits (Erickson et al. 2018; Roberts Kingman, Vyas, et al. 2021). We also find that genes with ASE are enriched on particular chromosomes (supplementary figs. S8 and S9, Supplementary Material online; see Methods). EcoPeaks and QTL associated with phenotypic divergence are particularly concentrated on ChrIV and this chromosome also harbored the highest proportion of ASE genes over background (Permutation *P<* 0.001) as well as a quarter of EcoPeak ASE genes (154 genes). A more modest enrichment of ASE genes was also found for chrXXI (*P* = 0.034) and chrXI (*P* = 0.033), which have been shown to harbor inversions between marine and freshwater fish (Jones et al. 2012). We identified 56 and 48 ASE genes within EcoPeaks on these chromosomes, respectively.

To identify potential candidate genes for marine-freshwater divergence, we overlapped ASE genes identified in marine-freshwater peaks with QTL for dental and skeletal traits (Cleves et al. 2014; Miller et al. 2014; Erickson et al. 2016) (figs. 4*C* and *D*). QTL for variation in dental traits between PAXB freshwater and marine fish (e.g., VTP or DTP tooth plate size and shape, tooth number, and jaw size and shape) overlapped 401 genes with ASE in relevant tissues (supplementary file S1, Supplementary Material online). A small subset of these have previously been implicated in dental or craniofacial morphology in other species (table 1), including several genes involved in the Wnt signaling pathway identified in the sign test (e.g., *Wnt5a*, *Sfrp2*, *Ctnnb1*, *Net1*).

**Table 1.** Candidate ASE Genes Within Differentiated Regions With Overlapping QTL.

Gene	Marine-Freshwater EcoPeak	ASE tissues	QTLs^a^	Relevant functions
*Cldn4*	chrI:16985780–17009145	DTP 1&2, VTP 1, Mandible 1, Liver	Tooth plate shape	Tooth development (Bello et al. 2007)
*Igfbp5a*	chrI:26093586–26548184	DTP 2	Tooth plate shape	Tooth development, bone development (Mukherjee and Rotwein 2008: 5; Aizawa et al. 2019: 3)
*Scube1*	chrIV:21368021–22019696	DTP 1, VTP 1, Mandible 2, Eyes	Tooth number, jaw shape	Craniofacial development (Xavier et al. 2009)
*Stc2a*	chrIV:13853040–14033725	Mandible 1	Jaw shape	Skeletal development (Johnston et al. 2010)
*Net1*	chrIV:21368021–22019696	DTP 1, VTP 1&2, Mandible 1&2, Flank skin	Tooth plate tooth number, jaw shape	Tooth development, bone development (Abdullah et al. 2021: 1)
*Kdm5a*	chrIV:26410831–26967766	VTP 1	Tooth plate tooth number	Tooth development (Li et al. 2020)
*Kdm6bb*	chrVII:19682650–19906375	DTP 1&2, VTP 1	Tooth plate area and shape, tooth plate tooth number, dentary shape	Tooth development, bone development (Xu et al. 2013; Zhang et al. 2015: 3)
*Cldnb*	chrVII:21853746–22015247	DTP 1&2, VTP 2, Mandible 1&2, Flank skin	Jaw, dentary, and tooth plate shape, tooth plate tooth number, defense plates	Tooth development (Bello et al. 2007)
*Postna*	chrVII:22876973–23002851	DTP 1&2, Flank skin, VTP 2, Mandible 1 & 2, eyes	Jaw shape, dentary shape, tooth plate shape, tooth plate tooth number, defense plates	Tooth development, bone development (Romanos et al. 2014)
*Kdm6ba*	chrVII:8555721–8746343	Mandible 1, Liver	Jaw shape	Bone development (Zhang et al. 2015)
*Timp2b*	chrXI:9651474–9924679	DTP 1&2, Flank skin, eyes, liver	Tooth plate tooth number	Tooth development (Nunia et al. 2016)
*Mmp9*	chrXII:10684220–10754257	DTP 2, Flank skin	Tooth plate shape	Tooth development, bone development (Vu et al. 1998; Ni and Chen 2014)
*Itga5*	chrXII:7057481–7113347	DTP 1	Tooth plate shape	Tooth development (Wang et al. 2019)
*Wnt5a*	chrXII:8202228–8410911	DTP 1, Flank skin, VTP 1&2, Mandible 1	Tooth plate shape, dentary shape	Tooth development, facial development (Lin et al. 2011; Hosseini-Farahabadi et al. 2017)
*Tgfbr1b*	chrXXI:3449938–3520071	VTP 1, DTP 2	Tooth plate area, tooth plate shape	Tooth development (Niwa et al. 2018)
*Sulf1*	chrXXI:9696109–11646044	DTP 1, VTP 1&2, Mandible 2, eyes	Tooth plate shape, jaw shape	Tooth development, skeletal development (Ratzka et al. 2008; Hayano et al. 2012)
*Bmi1a*	chrXXI:9696109–11646044	VTP 1	Tooth plate tooth number, tooth plate shape	Tooth development (Yin et al. 2021)
*Mllt10*	chrXXI:9696109–11646044	DTP 1&2, VTP 1& 2	Tooth plate shape, tooth plate tooth number	Craniofacial development (Ogoh et al. 2017)

a QTL data: Miller et al. (2014), Cleves et al. (2014), and Erickson et al. (2016).

## Conclusions

Changes in gene expression regulation are thought to play a major role in evolutionary adaptation. Here, we surveyed allele-specific expression across tissues and developmental stages to understand the landscape of *cis-*regulatory divergence between marine and freshwater sticklebacks. We identified widespread ASE that was largely heterogeneous between tissue types and developmental stages. For a subset of these *cis-*regulatory changes, we found evidence for polygenic selection on particular processes/pathways with a sign test. Finally, we demonstrated that *cis-*regulatory changes are often associated with regions of marine-freshwater divergence, further supporting the role of *cis*-regulatory differences in adaptive evolution in sticklebacks (Jones et al. 2012; Roberts Kingman, Vyas, et al. 2021).

Our results indicate that *cis*-regulatory divergence between marine and freshwater fish is often specific to an individual tissue or developmental stage. Gene expression differences that are spatially or temporally restricted may be important in the process of adaptation to new environments. Through context-specific expression regulation, *cis*-regulatory mutations can avoid negative pleiotropy associated with global changes in expression or protein structure. Thus, it is possible that *cis-*regulatory variation that introduces discrete changes in gene expression may be favored during adaptation. Interestingly, we found that genes with evidence for tissue-specific ASE, in particular, were enriched in regions of recurrent marine-freshwater divergence. Tissue- or context-specific *cis-*regulatory differences have previously been shown to underlie adaptive traits in sticklebacks (Miller et al. 2007; Chan et al. 2010) and other systems (Hu et al. 2022). The tissue- and developmental-specificity of *cis-*regulatory changes that we identified between marine and freshwater sticklebacks highlight the utility of studying gene regulation across multiple tissues and contexts in understanding regulatory adaptation.

Genes with ASE in regions of repeated marine-freshwater divergence may be interesting candidates for adaptive phenotypic differences between ecotypes. *Cis-*regulatory changes have been found to underlie a number of phenotypic differences between marine and freshwater forms. For example, *cis-*regulatory changes at *Bmp6* are associated with evolved tooth gain (Cleves et al. 2014; Stepaniak et al. 2021), and *cis*-regulatory changes at *Eda* and *GDF6* have been implicated in the skeletal differences between marine and freshwater fish (O’Brown et al. 2015; Indjeian et al. 2016). While these three genes did not have sufficient expression to reliably measure ASE in our data, we identified several interesting candidate ASE genes within differentiated genomic regions. For example, *cis*-regulatory variation at *Stanniocalcin2a* (*Stc2a*) was recently associated with changes in spine length in freshwater sticklebacks (Roberts Kingman, Lee, et al. 2021). We found that *Stc2a* also showed ASE in the early mandible timepoint. *Stc2a* falls within a marine-freshwater divergent region on ChrIV that overlaps several QTL, including the QTL with the largest effect on dentary size in crosses between PAXB freshwater fish and marine fish (Miller et al. 2014). In mice, *Stc2a* modulates bone size and growth, and overexpression results in smaller mandibles (Gagliardi et al. 2005; Johnston et al. 2010), making this gene an exciting candidate for divergent jaw morphology between marine and freshwater benthic fish. Our results also highlighted Wnt signaling genes as potential candidates for divergence in feeding morphology. A sign test indicated evidence for lineage-specific selection on *cis-*regulatory alleles involved in Wnt signaling, and several of these genes were also found within QTL/EcoPeaks and involved in tooth development or craniofacial morphology (see supplementary table S1, Supplementary Material online). For example, *Wnt5a,* associated with QTL for tooth plate and dentary shape, plays an important role in facial and tooth development in mammals (Yamaguchi et al. 1999; Lin et al. 2011; Hosseini-Farahabadi et al. 2017). Our results establish the landscape of stickleback *cis*-regulatory divergence across tissues and developmental stages; we look forward to future studies that elucidate the roles that specific ASE genes have played in stickleback adaptation.

## Methods

### Stickleback Husbandry

All animal work was approved by UC Berkeley IACUC protocol AUP-2015–01-7117. Fish were raised in aquaria at 18° C in brackish water (3.5 g/l Instant Ocean salt, 0.217 ml/l 10% sodium bicarbonate) with 8 h of light per day. Fry (SL < 10 mm) were fed live Artemia, whereas early juveniles (SL ∼10-29 mm) were fed live Artemia and frozen Daphnia. Fish above ∼20 mm were fed frozen bloodworms and Mysis shrimp. To generate F1 hybrids, a freshwater Paxton Benthic (Paxton Lake, Canada) strain male was crossed with a marine Rabbit Slough (Alaska) strain female. Individuals from these lineages have been maintained in the lab for >10 generations. The resulting full-sibling fish were raised together in a common dish or tank until sample collection. Female F1 hybrids were selected for dissection at two timepoints (15–20 mm SL and 35 mm SL). Fish were euthanized individually via immersion in 250 mg/l MS-222. Tissue samples for RNA-seq were immediately dissected on an ice-cold tray. Brain samples included all bilateral brain regions from the olfactory bulb to the brain stem. Liver samples were derived from the anteriormost lobe of the fish liver. Eye samples encompassed the entirety of the left eye of each fish, including the majority of the optic nerve. Flank skin samples were taken by removing the majority of the skin covering the left side of each fish, capturing a region that would normally be covered by lateral armor plates in adulthood (anteriormost boundary at the level of the 1st dorsal spine, where the anteriormost armor plates had begun ossification at the time of dissection, and posterior boundary at the back of the dorsal fin where armor plates were not yet ossified). Dorsal pharyngeal tooth plate samples included left and right DTP1 and DTP2, as well as underlying epibranchial bones and surrounding soft tissues and teeth. Ventral pharyngeal tooth plate samples included left and right ceratobranchial 5 and surrounding soft tissues and teeth. The mandible consisted of the dentary bone and lower lip, and all associated soft tissues and teeth. Samples were placed into 50 µl of TRIzol (Invitrogen), briefly agitated by shaking, and incubated on ice for 10 min. All samples from each timepoint were prepared on the same day.

### RNA Extraction, Library Preparation, and Sequencing

Dissected tissues were kept in TRI reagent and stored at −80° C prior to RNA-extraction. Total RNA extraction was performed as described previously (Hart et al. 2018). Total RNA was quantified by a Qubit Fluorometer, and quality was checked by an Agilent Bioanalyzer. Libraries were constructed with New England Biolabs NEBNext Poly(A) mRNA Magnetic Isolation Module (E7490S), NEBNext Ultra II Directional RNA Library Prep Kit (E7765S), and NEBNext Multiplex Oligos for Illumina (96 Unique Dual Index Primer Pairs, E6440S) following the manufacturer's instructions. Library quality was analyzed on an Agilent Bioanalyzer (supplementary table S1, Supplementary Material online). Libraries were pooled and sequenced on an Illumina HiSeq platform (2 × 150 bp reads). We obtained a total of 1,439,700,457 reads across 20 samples (10 tissues × 2 replicates) (supplementary table S1, Supplementary Material online).

### Whole-genome re-sequencing of PAXB

To phase RNA-seq reads, whole-genome resequencing was performed on the PAXB parent. DNA was extracted from fin tissue. Library preparation and sequencing were performed by Admera Health (South Plainfield, NJ). Libraries were sequenced on an Illumina HiSeqX platform (2 × 150 bp reads) to a depth of ∼30 × (supplementary fig. S1, Supplementary Material online). Coverage per site was calculated with Samtools depth (Li et al. 2009) based on reads aligned to the reference genome (described below).

### Read Mapping and SNP Calling

RNA-seq read quality was assessed using FastQC. Reads were trimmed for adaptor sequences with Trimmomatic (Bolger et al. 2014) and then mapped to the stickleback reference genome (Peichel et al. 2017). F1 hybrid RNA-seq reads were mapped to the stickleback reference genome with STAR v2.7 (Dobin et al. 2013). Genomic reads from PAXB were mapped with bowtie2 v2.3.4 (argument: –very-sensitive) (Langmead and Salzberg 2012).

SNP calling was then performed with the Genome Analysis Tool Kit (GATK) (McKenna et al. 2010). Duplicates were marked with the Picard tool MarkDuplicates. Read groups were added with AddOrReplaceReadGroups. For RNAseq reads, we used GATK tool SplitNCigarReads to split reads that contain Ns in their cigar string (e.g., spanning splice events). GATK HaplotypeCaller and GenotypeGVCFs were used for joint genotyping. SNP calls were subsequently filtered for low quality calls with VariantFiltration (QD < 2.0; QUAL < 30.0; FS > 200; ReadPosRankSum < −20.0).

To assign allele-specific reads to the parent of origin (i.e., “freshwater” parent vs. “marine” parent), we retained only variants where the PAXB parent was homozygous. Heterozygous sites for each F1 individual were used for separating allele-specific RNA-seq reads into freshwater and marine pools, as described below.

### Identifying Allele-specific Expression

To identify allele-specific expression (ASE), reads from each library were then mapped again with STAR, implementing the WASP filter based on heterozygous calls (van de Geijn et al. 2015). WASP reduces mapping bias by identifying reads containing SNPs, simulating reads with alternative alleles at that locus, re-mapping these reads to the reference, and then flagging reads that do not map to the same location. Reads that do not map to the same location were discarded (van de Geijn et al. 2015). Parental origin for each allele was assigned based on PAXB (freshwater parent, see above). Reads were counted over marine-freshwater variants with ASEReadCounter (McKenna et al. 2010) for individual heterozygous sites. To mitigate the effects of SNP calling errors and read mapping bias, we removed heterozygous sites with (1) large ratio differences indicative of mapping bias (log_2_ fold changes of allelic counts > 10), or (2) no reads mapped to one of the parental alleles. Mapping was then repeated a second time based on the updated list of heterozygous sites. Analysis of ASE ratios in each library centered around a log_2_ ratio of zero, indicating approximately equal mapping to both parental alleles.

Gene-wise estimates of allele-specific expression were quantified by counting allele-specific reads overlapping exons using HTSeq (Anders et al. 2015) based on Ensembl annotations (BROAD S1) (Jones et al. 2012), with coordinates converted by LiftOver to the v4 stickleback assembly (https://stickleback.genetics.uga.edu/downloadData/) (Peichel et al. 2017). Total counts per parental allele per tissue are available in supplementary table S2, Supplementary Material online. Across tissues, we did not observe a consistent bias toward either of the parental alleles. To examine transcriptome-wide patterns of expression, we transformed expression values (allele-specific and total counts) using variance stabilizing transformation and assessed the patterns via principal components analysis (PCA) (fig. 1*B*, supplementary fig. S2, Supplementary Material online).

DESeq2 (Love et al. 2014, p. 2) was used to identify ASE using the individual as a blocking factor and allele-specific expression (“marine” vs. “freshwater” allele) as the variable of interest (Wald test). As read counts from “marine” and “freshwater” alleles come from the same sequencing library, library size factor normalization was disabled by setting SizeFactors = 1. *P*-values were adjusted using the Benjamini–Hochberg method in DESeq2 for multiple comparisons. Genes were examined at FDR < 0.05 and FDR < 0.1 (supplementary table S3, Supplementary Material online). Comparing genes with ASE in VTP from the late timepoint with the results of Hart et al. (2018), which also tested for ASE in crosses between PAXB and RABS in the VTP, we found a highly significant overlap (Fisher's exact test, *P* = 8.83 × 10^−292^). Fifty-one percent of ASE genes identified here were also identified in the previous analysis. Additionally, log_2_ fold changes of genes with ASE were found to be correlated (Pearson's Correlation, *r* = 0.64, *P* = 2.47 × 10^−69^). Segregating variation could potentially lead to the inference of tissue-specific expression when different individuals are sampled for different tissues. However, tissues obtained from different individuals were not found to have a greater proportion of tissue-specific genes compared with tissues sampled from the same individuals (supplementary table S10, Supplementary Material online). We also repeated our ASE analysis using only sites genotyped as heterozygous in all F1 hybrids for which we had coverage. DESeq2 *P*-values were highly correlated across analyses (supplementary table S11, Supplementary Material online).

As in the previous analysis, tissue-specific ASE was found to be a predominant source of regulatory variation (46% of genes at the second timepoint) and tissues showed similar allelic expression patterns based on PCA (supplementary fig. S10, Supplementary Material online).

Developmental stage differences in ASE were identified by comparing reads mapping to freshwater versus marine alleles at both timepoints, summed across the two replicates. We compared marine and freshwater allelic ratios for genes with evidence of ASE in at least one of the two developmental stages with a Fisher's exact test. The resulting *P*-values were corrected using the Benjamini–Hochberg method.

### Assessing Heterogeneity in Allele-Specific Expression Across Tissues

To identify heterogeneity in allele-specific expression across tissues, we adopted a Bayesian model comparison framework from Pirinen et al. (2015). In this approach, tissues are classified as no ASE (θ(N)), strong ASE (θ(S)), or moderate ASE (θ(M)) based on freshwater and marine allelic counts summed across replicates per gene under a grouped tissue model (Pirinen et al. 2015). Tissues are further classified as showing ASE heterogeneity if one tissue showed evidence for either strong or moderate ASE and at least one other tissue did not show ASE (HET0), or when all tissues showed some evidence for ASE but the magnitude differed (HET1). Finally, we consider a substate of ASE heterogeneity tissue-specificity, where the ASE state (i.e., moderate, strong ASE, or no ASE) is observed in only one tissue (Pirinen et al. 2015).

The following priors were selected to describe groups:

θ(N)∼Beta(2000,2000)
θ(M)∼ ½Beta(80,36)+ ½Beta(36,80)
θ(S)∼ ½Beta(80,7)+½Beta(7,80)

Densities of the prior distributions for the proportion of allelic counts are found in supplementary figure S11, Supplementary Material online. Parameters for Beta distributions were chosen to clearly separate the three groups from each other to allow the classification of tissues to a particular group, following Pirinen et al. (2015). The “No ASE” condition dominates the region around 0.5 (0.47,0.53), allowing some deviation for technical bias or noise (Skelly et al. 2011; Pirinen et al. 2015). Strong ASE dominates at extreme frequencies ([0.85,0.96], [0.3,0.15]) and moderate ASE dominants between these two groups (supplementary fig. S11, Supplementary Material online). Genes expressed in at least two tissues at a minimum depth of 10 reads/allele were included in the analysis (15,477 genes). For each gene, we excluded tissues for which coverage was low (less than or equal to 10 reads per allele).

### Sign Test on ASE

To search for selection on *cis*-regulatory variation, we applied a sign test based on the directionality of ASE in a gene set (Bullard et al. 2010; Fraser et al. 2011). Gene Ontology (GO) categories for zebrafish were obtained from ZFIN (https://zfin.org/downloads) (Bradford et al. 2022) and mapped to stickleback orthologs based on Ensembl ortholog annotations. Genes with evidence for *cis-*regulatory divergence were divided into categories based on the upregulated allele (freshwater vs. marine). We excluded GO categories with fewer than 10 members in a test set with ASE. As test sets contain different proportions of upregulated marine versus freshwater alleles, we tested for lineage-specific bias in each test set with a Fisher's exact test.

Because many GO categories were tested, we determined the probability of an enrichment by permuting gene category assignments, as described previously (Bullard et al. 2010; Fraser et al. 2011; Artieri et al. 2017). Gene assignments were shuffled and the test was repeated 10,000 times. Permutation-based *P*-values were determined by asking how often a result of equal or greater significance would be observed in permuted datasets (Bullard et al. 2010; Fraser et al. 2011; Artieri et al. 2017). Tests were performed on individual tissues and on the dental tissues together, as many genes with ASE are shared across these tissues. In the grouped tissue analysis, we looked for biased directionality across all genes with ASE in a tissue group. In the event that signs differ between tissues (i.e., freshwater allele is upregulated in tissue #1, marine allele is upregulated in tissue #2), the gene is discarded from the analysis. Changes in directionality across tissues was only seen for one gene associated with a significant GO term (dental tissue: “inflammatory response”). To ensure that biased directionality in our group analyses were robust to tissue-specific patterns, we performed a second test, where we combined *P*-values for a tissue group from individual tissues with Fisher's method, as in (Fraser et al. 2011). We performed a Fisher's exact test for each category as described above for individual tissues. *P-*values for GO categories that are represented across all tested groups were then combined using the R package metap. We report on the GO terms significant in both approaches, as these represent cases of biased directionality across tissue groups and are robust to individual tissue patterns. Combined *P*-values are reported in supplementary table S6, Supplementary Material online.

### Identifying Overlap With EcoPeaks

Data from Roberts Kingman, Vyas, et al. (2021) were downloaded from the UCSC Genome Browser Table Browser. Intervals were associated with overlapping genes using bedtools (Quinlan and Hall 2010). As power to detect ASE is related to the density of informative sites, we calculated SNP density per gene as the number of informative heterozygous sites divided by transcript length, based on BROAD S1 gene annotations (Roberts Kingman, Vyas, et al. 2021). To determine whether genes with ASE had higher average Z-scores than background genes, while controlling for the effect of SNP density on our power to identify ASE, we grouped genes with similar SNP densities into bins based on the distribution of SNP density values. We excluded bins for which there were fewer than five genes in each category (ASE, no ASE), so as not to skew results based on bins with few observations (330 bins, average of 23 genes per bin) (supplementary fig. S8*A*, Supplementary Material online). For each bin, we calculated the median Z-score for genes with ASE and background genes (supplementary fig. S8*B*, Supplementary Material online). We compared this result to a permuted dataset. Within a density bin, we shuffled the gene category assignments and again calculated the median Z-score for each category in each bin. We repeated this 10,000 times. To obtain a permutation-based *P*-value, we compared how often the median difference in category Z-scores was as extreme or more extreme than in empirical data. This result was robust to varying bin sizes (supplementary table S12, Supplementary Material online).

To ask whether ASE genes were enriched on particular chromosomes, as with QTLs and EcoPeaks (Roberts Kingman, Vyas, et al. 2021), we performed a resampling test to account for differences in SNP density between genes. We sampled random sets of genes (equal to the number of total ASE genes) with SNP densities matched to the ASE gene set (1,000 times). The number of genes associated with each chromosome was counted for each permuted gene set and compared with the empirical data. *P*-values were calculated based on how often an equal or more extreme result was observed for permuted gene sets (supplementary fig. S9, Supplementary Material online).

### Gene Annotations and QTL Overlap

Stickleback genes were annotated to zebrafish and mouse orthologs based on Ensembl ortholog annotations. BiteCode genes were annotated as in Hart et al. (2018), from the BiteIt database (http://bite-it.helsinki.fi/) and ToothCODE database (http://compbio.med.harvard.edu/ToothCODE/). Phenotype annotations for zebrafish were downloaded from ZFIN (https://zfin.org/downloads), and mouse mutant phenotypes were downloaded from Ensembl and the Mouse Genome Database (Blake et al. 2021).

QTL coordinates for overlap are based on genomic coordinates in Peichel and Marques (2017). For overlap with ASE genes, we focused on QTL mapping studies utilizing crosses between PAXB freshwater and marine individuals. Dental QTL for supplementary table S1, Supplementary Material online, and supplementary file S1, Supplementary Material online were obtained from three studies (Cleves et al. 2014; Miller et al. 2014; Erickson et al. 2016). Coordinates were converted by LiftOver to the v4 stickleback assembly for overlap with EcoPeaks. Genes of interest for table 1 were identified based on intersections between these genes (ASE/EcoPeak/QTL) and phenotype/Gene Ontogony annotations or the BiteCode gene list. A full list of gene overlaps is available in supplementary file S1, Supplementary Material online.

## Supplementary Material

msad034_Supplementary_Data

## Data Availability

All sequence data generated in this study have been deposited to the National Center for Biotechnology Information Sequence Read Archive as a BioProject (PRJNA887031). Supplemental datasets are available in supplementary file S1, Supplementary Material online. Scripts associated with this manuscript are available on GitHub (https://github.com/katyamack-hub/SticklebackASE).
