# Supplementary material for: Evolution of Spatial and Temporal cis-Regulatory Divergence in Sticklebacks
Source: Mol Biol Evol. 2023 Feb 20;40(3):msad034. doi: 10.1093/molbev/msad034 (PMC10015619; doi:10.1093/molbev/msad034)
Supplement: msad034_Supplementary_Data [file msad034_supplementary_data.zip › Mack_SticklebackASE_SupplementalMaterial_Figures_Tables_v3_MBE_Revision_2_2_23_v2.pdf]

Supplemental Material

Supplemental Figures

Figure S1. Coverage of PAXB genome re-sequencing.

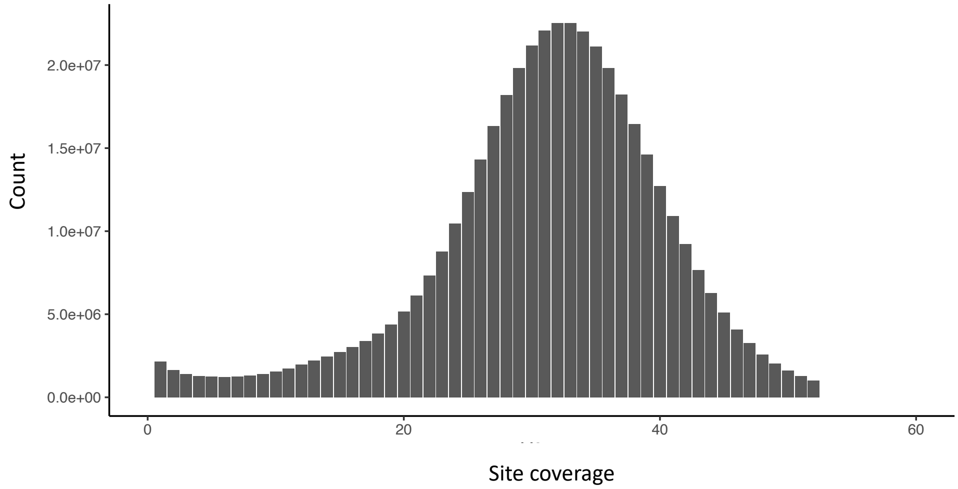

Figure S2. PCA of total RNAseq reads across tissues of marine-freshwater F1s.

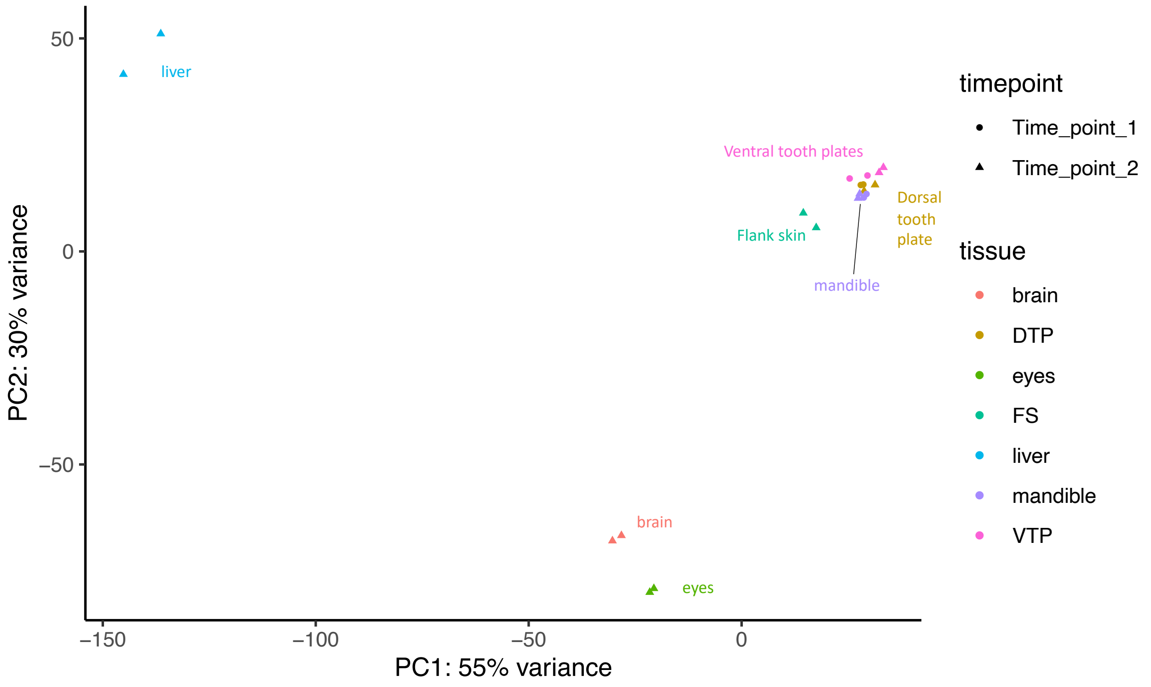

Figure S3: PCA of dental tissues from two developmental stages. Shape indicates freshwater (triangle) vs. marine allele (circle) and color indicates developmental stage (time 1 = red, time 2 = teal).

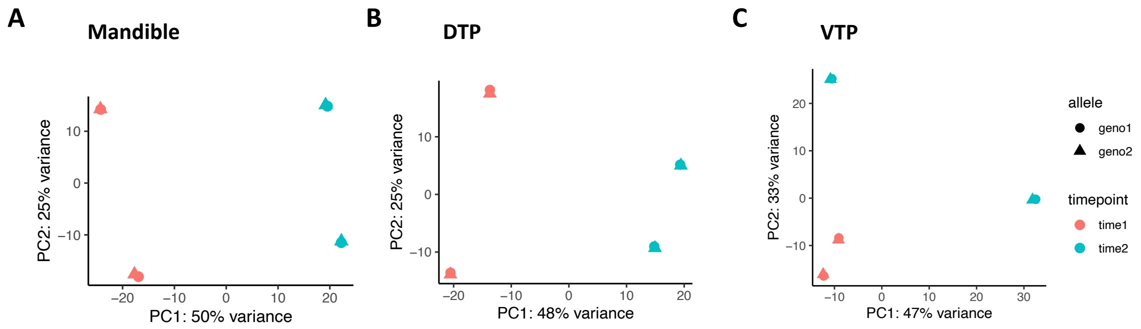

Figure S4. Total allele-specific reads per tissue vs. number of genes with evidence for ASE in a tissue (at FDR<0.05)**.** Each dot represents a tissue. Allele-specific read depth was not correlated with number of ASE genes identified in a tissue (Spearman's *rho* = -0.24, *p* = 0.56).

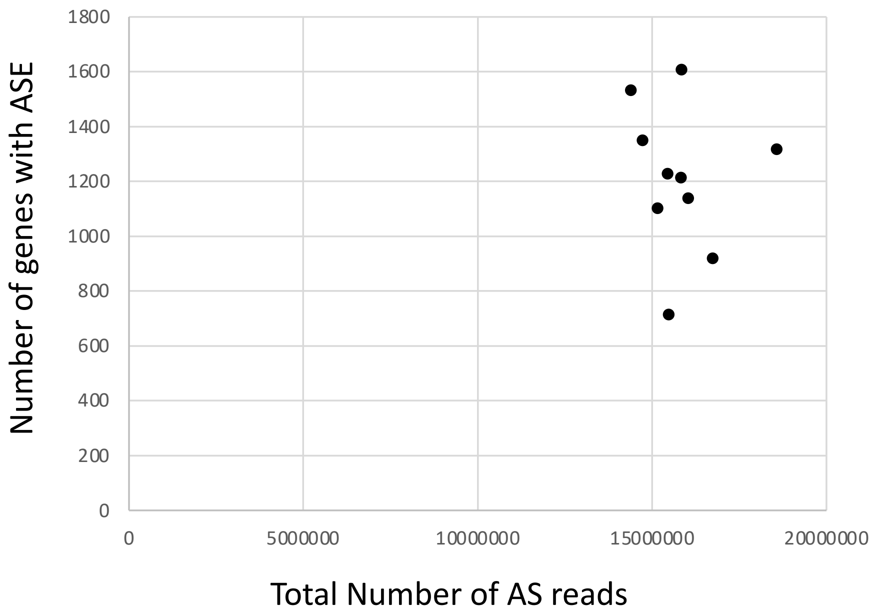

Figure S5. A. PCA of average log2 fold change (marine/freshwater allele) of dental tissues. **B**. BiteCode genes with ASE. Genes are colored by their log2 fold change. Asterisks indicate significance level *FDR<0.05, **FDR<0.01, ***FDR<0.001.

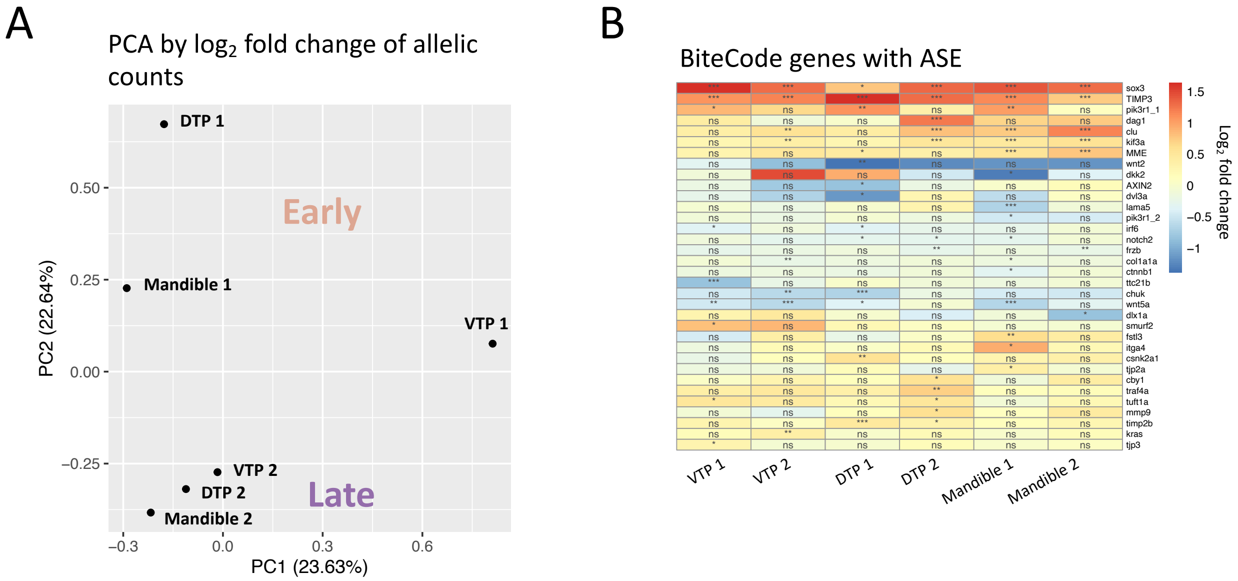

Figure S6. UpSet plot of overlap of ASE across dental tissues at two developmental stages.

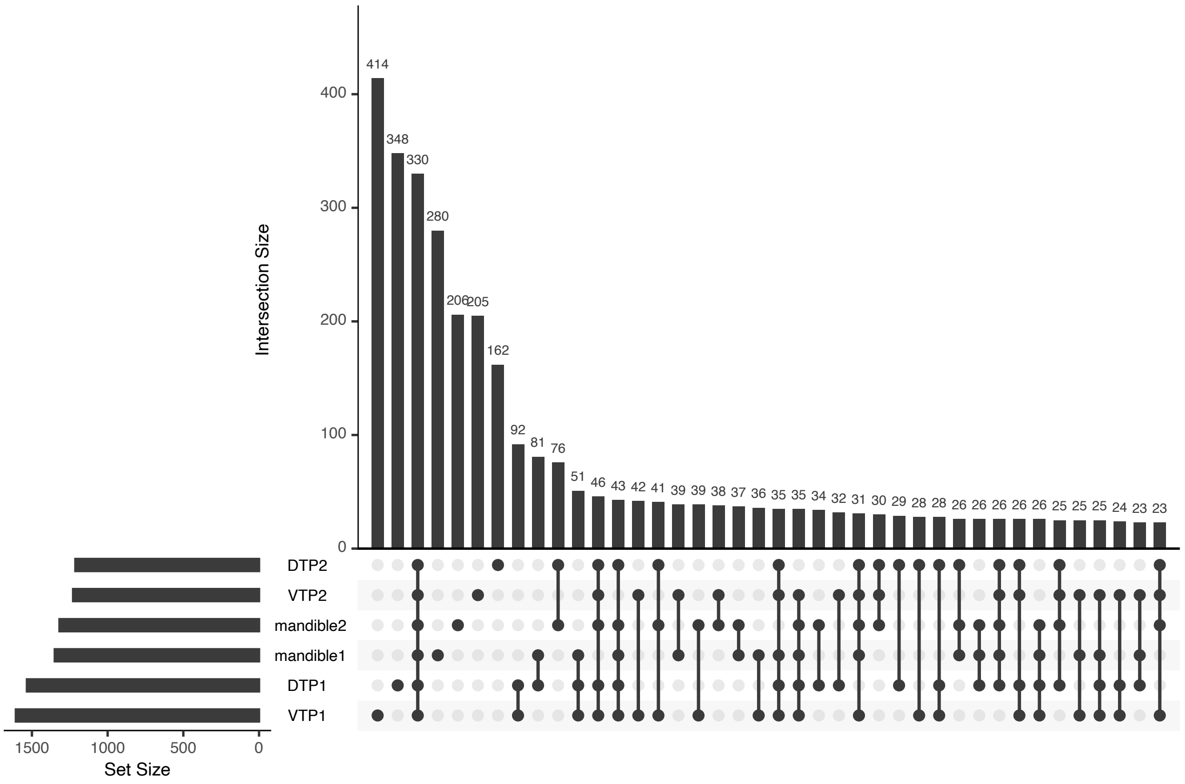

Figure S7. A. Density plots of average SNP density in bins for ASE genes (right) and background genes (left). A similar density distribution is seen for ASE vs. background for bins. **B.** Histogram of median Z-scores of ASE genes and background genes for bins (bars are interleaved for visualization). **C.** Gene median Z scores vs. bin. Each point is a gene.

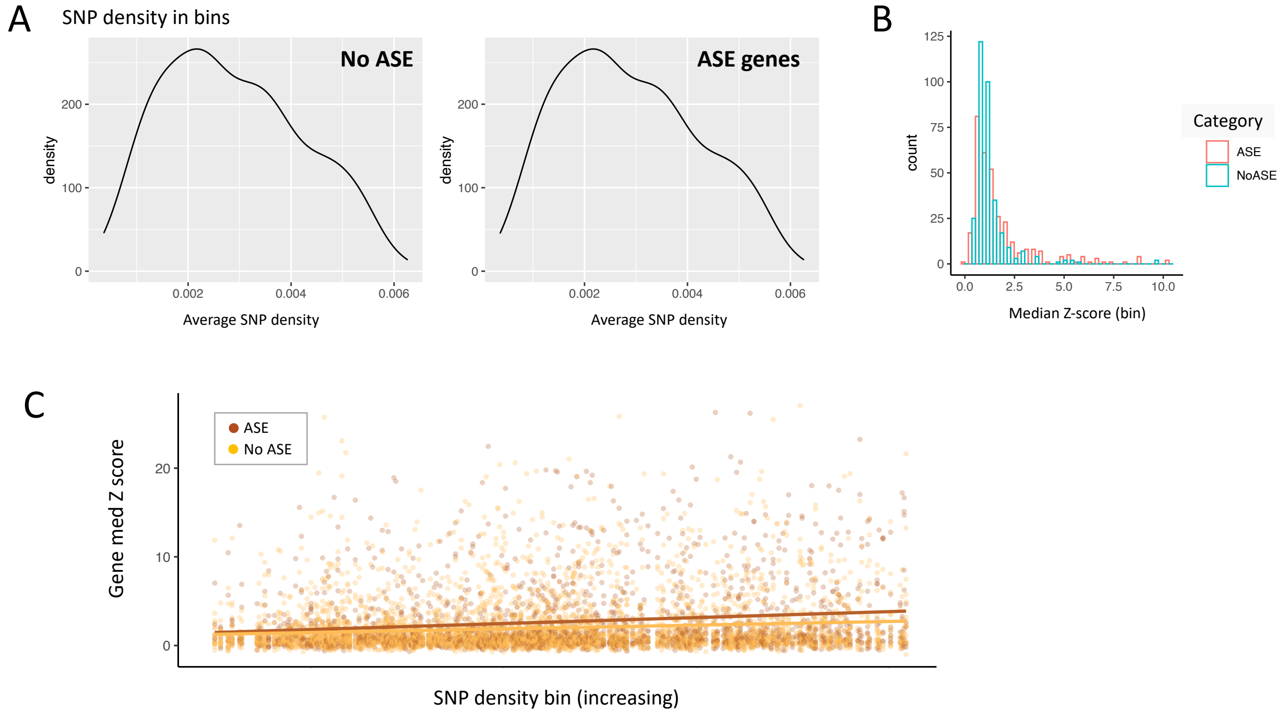

Figure S8. Proportion of gene on each chromosome with ASE for each tissue (number of genes with ASE/background on a chromosome)

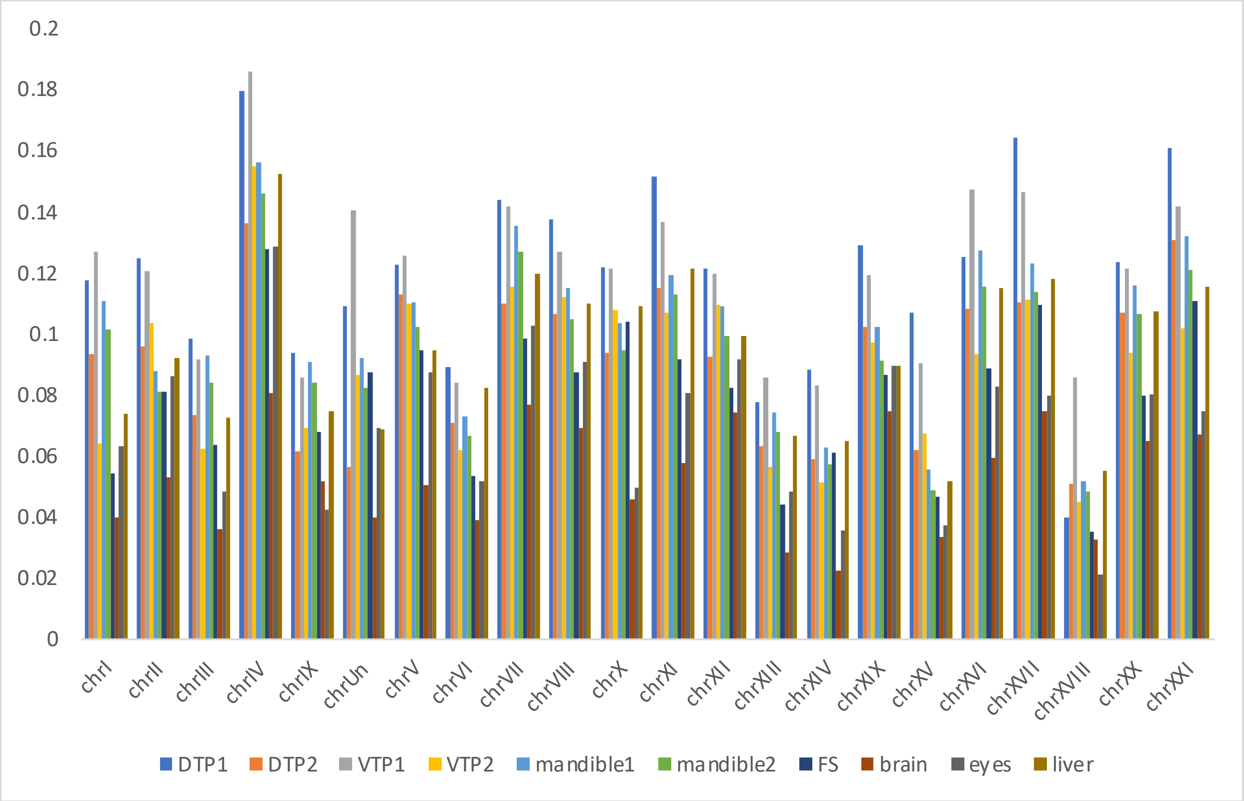

Figure S9. Chromosomes with an enrichment of ASE over background expression (*P*-values reported based on permutation).

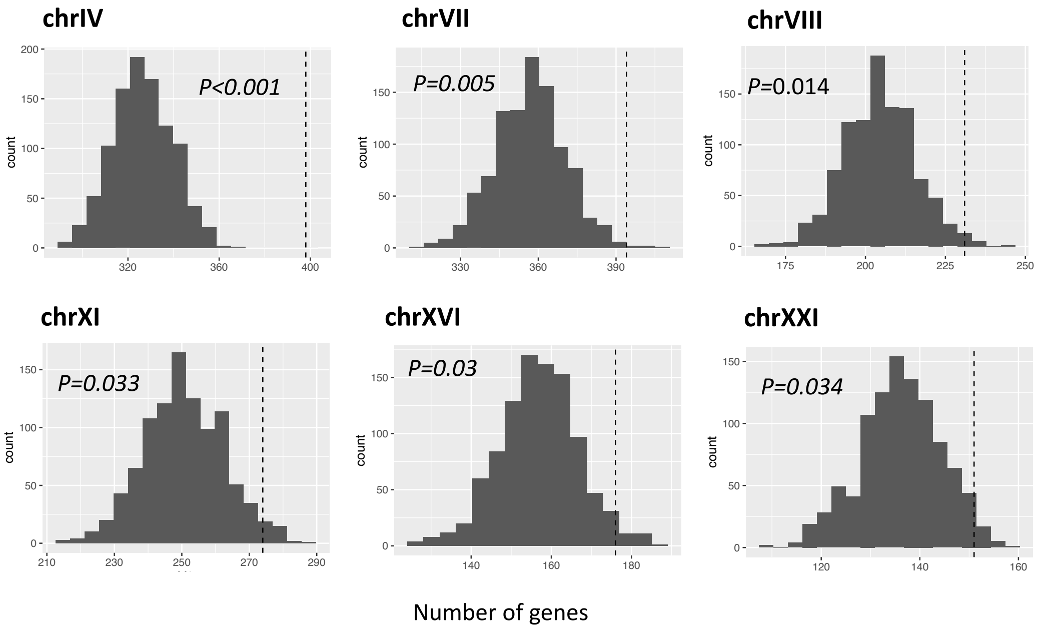

Figure S10. PCA of allelic expression limited to sites genotyped as heterozygous across F1 samples. Samples are colored by tissue. Shape indicates allele (marine allele = circle, freshwater = triangle).

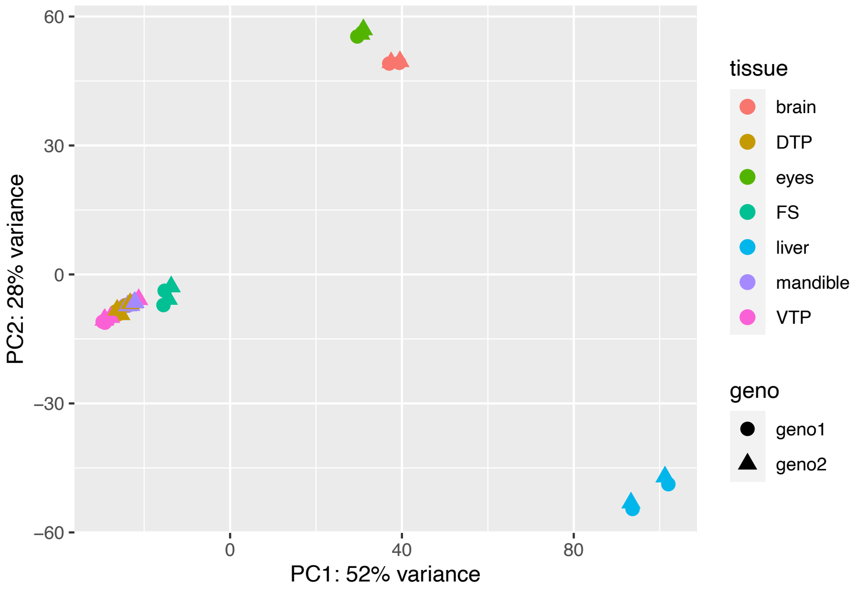

Figure S11. Densities of the prior distributions for the proportion of the freshwater allele. Parameters for Beta distributions were chosen to clearly separate the three groups from each other to allow the classification of tissues to a particular group (no ASE ($\theta(\mathcal{N})$), strong ASE ($\theta(\mathcal{S})$), or moderate ASE ($\theta(\mathcal{M})$)), following Pirinen et al. (Pirinen et al. 2015).

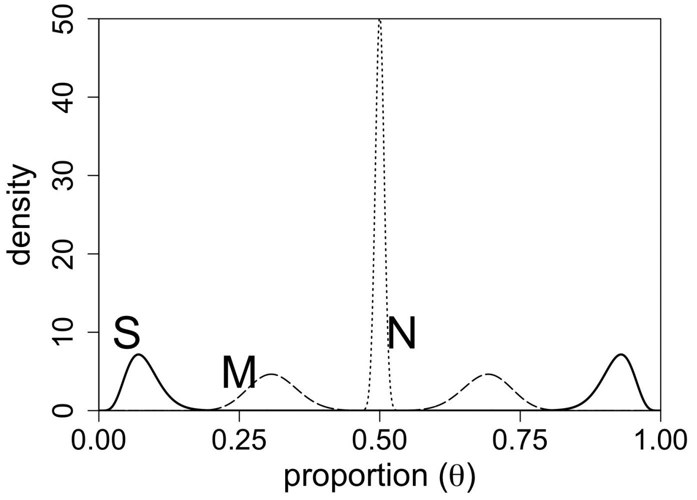

Table S1. RNAseq library sample information

Sample_ID	Tissue (individual ID)	Timepoint	RIN score	Raw reads	Mapped Reads (total)
18146FL-37-01-01_S74	Brain (5)	Time point-2	9.8	83303949	75363055
18146FL-37-01-02_S75	Brain (6)	Time point-2	10	70737373	63643543
18146FL-37-01-05_S78	DTP (1)	Time point-1	7.7	66748054	57054598
18146FL-37-01-06_S79	DTP (2)	Time point-1	8	66666757	58252092
18146FL-37-01-03_S76	DTP (7)	Time point-2	9.5	82208758	73258066
18146FL-37-01-04_S77	DTP (8)	Time point-2	8.9	61076908	54068495
18146FL-37-01-07_S80	Eyes (5)	Time point-2	9	66645858	59796162
18146FL-37-01-08_S81	Eyes (6)	Time point-2	10	91056685	82687823
18146FL-37-01-09_S82	FS (5)	Time point-2	9.8	62002949	54244891
18146FL-37-01-10_S83	FS (6)	Time point-2	10	69199627	61208309
18146FL-37-01-11_S84	Liver (5)	Time point-2	10	66280457	60093436
18146FL-37-01-12_S85	Liver (6)	Time point-2	9.8	66475414	59547137
18146FL-37-01-13_S86	Mandible (1)	Time point-1	7.4	63449422	55541248
18146FL-37-01-14_S87	Mandible (2)	Time point-1	8.7	65477982	57454455
18146FL-37-01-15_S88	Mandible (7)	Time point-2	10	99370852	87241465
18146FL-37-01-16_S89	Mandible (8)	Time point-2	10	65309621	58272877
18146FL-37-01-17_S90	VTP (4)	Time point-1	10	71780930	62100619
18146FL-37-01-19_S92	VTP (5)	Time point-2	7.9	92388481	81080118
18146FL-37-01-20_S93	VTP (6)	Time point-2	7.9	52927806	46928285
18146FL-37-01-18_S91	VTP (3)	Time point-1	8.1	76592574	64309864

Table S2. Allele-specific mapping depth per sample.

Individual	Freshwater allele	Marine allele
DTP_1, replicate 1	3745530	3685882
DTP_1, replicate 2	3473514	3474894
DTP_2, replicate 1	4692653	4688887
DTP_2, replicate 2	3211629	3216112
FS_2, replicate 1	3686618	3524350
FS_2, replicate 2	4003882	3930078
VTP_1, replicate 1	3956071	3901128
VTP_1, replicate 2	3984927	3982959
VTP_2, replicate 1	4836454	4777676
VTP_2, replicate 2	2917434	2897866
brain_2, replicate 1	4238704	4267511
brain_2, replicate 2	3452370	3501538
eyes_2, replicate 1	4751740	4821184
eyes_2, replicate 2	3542170	3604704
liver_2, replicate 1	4144471	4127935
liver_2, replicate 2	3917175	3829309
mandible_1, replicate 1	3590991	3473673
mandible_1, replicate 2	3876991	3769969
mandible_2, replicate 1	5617035	5452744
mandible_2, replicate 2	3820083	3673342

Table S3. Genes with ASE in each tissue

Tissue	FDR<0.05	FDR<0.1
Brain	714	963
Flank skin	1103	1424
Eyes	918	1194
Liver	1139	1423
Ventral toothplate (time 1)	1606	2088
Dorsal toothplate (time 1)	1533	1986
Mandible (time 1)	1349	1739
Ventral toothplate (time 2)	1228	1590
Dorsal toothplate (time 2)	1213	1579
Mandible (time 2)	1318	1603

Table S4. Pairwise overlap of genes with ASE between tissues (percentages in parentheticals correspond to [number of genes with ASE in both tissues]/[total Genes with ASE in across tissues])

	Brain	Eyes	Mandible	Liver	DTP	VTP	Flank skin
Brain							
Eyes	376 (29%)						
Mandible	314 (18%)	365 (19%)					
Liver	270 (17%)	340 (20%)	368 (18%)				
DTP	329 (21%)	380 (21%)	777 (44%)	371 (19%)			
VTP	343 (21%)	394 (22%)	676 (36%)	391 (20%)	665 (37%)		
Flank Skin	304 (20%)	370 (22%)	606 (33%)	372 (20%)	568 (36%)	611 (32%)	

Table S5. GO terms with biased directionality in single tissue analysis

Tissue	Biased term[1]	Permutation *P*
Eyes	methyltransferase activity (10/10)	0.0018
Mandible 1	peptidase activity (22/27)	0.0145
Mandible 1	fatty acid metabolic process (10/11)	0.0041
Flank skin	endoplasmic reticulum (38/50)	0.0039

[1]In parentheses are the number of terms in the same direction out of total

Table S6. GO terms with biased directionality in combined test of dental tissue

GO Term	# across tissues[1]	Permutation *P*	Fisher's combined *P*
canonical Wnt signaling pathway	9/10	0.0078	0.0012
inflammatory response	10/12[2]	0.0095	0.0086
embryonic viscerocranium morphogenesis	13/16	0.0093	0.02

[1]Genes in the same direction/the total number of genes with directionality

[2]In inflammatory response, one gene (*mpx*) switches direction between tissues (favoring the opposite allele)

Table S7. Canonical Wnt signaling genes with ASE in dental tissues

Gene Name	Pathway	Known role in tooth development
frzb	negative regulation of canonical Wnt signaling pathway (GO:0090090); canonical Wnt signaling pathway (GO:0060070)	Yes (Mitsiadis et al. 2017)
lmbr1l	negative regulation of canonical Wnt signaling pathway (GO:0090090)	
gsk3ab	negative regulation of canonical Wnt signaling pathway (GO:0090090)	Yes (Neves et al. 2017)
dab2	negative regulation of canonical Wnt signaling pathway (GO:0090090)	
sfrp2	negative regulation of canonical Wnt signaling pathway (GO:0090090); canonical Wnt signaling pathway (GO:0060070)	Yes (Wang et al. 2021: 2)
dkk2	negative regulation of canonical Wnt signaling pathway (GO:0090090)	Yes (Fjeld et al. 2005)
csnk1a1	negative regulation of canonical Wnt signaling pathway (GO:0090090)	
ctnnb1	canonical Wnt signaling pathway (GO:0060070)	Yes (Liu et al. 2008)
wnt5a	canonical Wnt signaling pathway (GO:0060070)	Yes (Lin et al. 2011)
dixdc1a	canonical Wnt signaling pathway (GO:0060070)	
net1	canonical Wnt signaling pathway (GO:0060070)	Yes (Løes et al. 2003)
mllt10	canonical Wnt signaling pathway (GO:0060070)	
dvl1a	canonical Wnt signaling pathway (GO:0060070)	
dvl3a	canonical Wnt signaling pathway (GO:0060070)	
wnt2	canonical Wnt signaling pathway (GO:0060070)	

Table S8. Genes associated with term “embryonic viscerocranium morphogenesis” with ASE in dental tissues.

Gene symbol	Gene Name
sar1b	secretion associated, Ras related GTPase 1B
ece1	endothelin converting enzyme 1
dlx1a	distal-less homeobox 1a
eif3ea	eukaryotic translation initiation factor 3, subunit E, a
dlx3b	distal-less homeobox 3b
gfpt1	Glutamine--fructose-6-phosphate transaminase 1
itga5	integrin, alpha 5 (fibronectin receptor, alpha polypeptide)
nbas	NBAS subunit of NRZ tethering complex
sphk2	sphingosine kinase 2
tmem165	transmembrane protein 165
hmgcrb	3-hydroxy-3-methylglutaryl-CoA reductase b
furina	furin (paired basic amino acid cleaving enzyme) a
nr3c1	nuclear receptor subfamily 3, group C, member 1 (glucocorticoid receptor)
polr1a	RNA polymerase I subunit A
ponzr1	plac8 onzin related protein 1
gnptab	N-acetylglucosamine-1-phosphate transferase subunits alpha and beta

Table S9. Genes overlapping EcoPeaks[1]

	ASE genes	All genes with expression	Total Genes
NE Pacific EcoPeaks (specific)	611	1201	1625
NE Pacific EcoPeaks (sensitive)	1534	3382	4736

[1]Genes are considered overlapping if the reported EcoPeak interval and gene overlap by 1 or more bp; overlap with NE "specific" peaks are discussed in main text

Table S10. Tissue-specific ASE genes across tissues

Tissue	Individuals	Tissue specific, Bayesian partitioning[1]	Tissue specific, presence/absence (DESeq2)[2]
Liver	5,6	141	366 (11,723)
Brain	5,6	66	102 (12,818)
DTP 2	7,8	24	115 (12,819)
VTP 2	5,6	30	131 (13,184)
Mandible 2	7,8	42	141 (13,553)
Flank skin	7,8	29	154 (13553)
eyes	5,6	85	171 (12,453)
DTP 1	1,2		274 (12,452)
VTP 1	3,4		343 (13,184)
Mandible 1	1,2		210 (12,452)

[1]Analysis based on expression across tissues at timepoint 2

[2]Significant ASE (DESeq2, FDR<0.05) in only one tissue-timepoint; total genes analyzed shown in parentheses

Table S11. Correlation of DESeq2 *p*-values between F1 genotyping approaches.

Tissue	*Rho*[1]
Liver	0.79
Brain	0.74
DTP 2	0.8
VTP 2	0.79
Mandible 2	0.8
Flank skin	0.78
eyes	0.74
DTP 1	0.79
VTP 1	0.72
Mandible 1	0.79

[1]Spearman's *rho*. All comparisons are significant at $p < 2.2 \times 10^{-16}$.

Table S12. SNP density bin sizes for comparisons between ASE and CSS Z-scores

Cuts	Number with sufficient genes[1]	Average number of genes	*P*-value[2]
200	200	66	3.72E-10
400	376	33	5.38E-16
500	430	26	1.09E-18
1000	400	13	1.59E-17

[1]Bins with at least 5 ASE genes and background genes

[2]*P*-values are based on a Wilcoxon signed-rank test comparing bin medians (ASE vs. background); all bins were significant via permutation test (*P*<0.001).

References

Fjeld K, Kettunen P, Furmanek T, Kvinnsland IH, Luukko K. 2005. Dynamic expression of Wnt signaling-related Dickkopf1, -2, and -3 mRNAs in the developing mouse tooth. Dev Dyn. 233(1):161–166. doi:10.1002/dvdy.20285.

Lin M, Li L, Liu C, Liu H, He F, Yan F, Zhang Y, Chen Y. 2011. Wnt5a regulates growth, patterning, and odontoblast differentiation of developing mouse tooth. Dev Dyn. 240(2):432–440. doi:10.1002/dvdy.22550.

Liu F, Chu EY, Watt B, Zhang Y, Gallant NM, Andl T, Yang SH, Lu M-M, Piccolo S, Schmidt-Ullrich R, et al. 2008. Wnt/β-catenin signaling directs multiple stages of tooth morphogenesis. Developmental Biology. 313(1):210–224. doi:10.1016/j.ydbio.2007.10.016.

Løes S, Luukko K, Hals Kvinnsland I, Salminen M, Kettunen P. 2003. Developmentally regulated expression of Netrin-1 and -3 in the embryonic mouse molar tooth germ. Developmental Dynamics. 227(4):573–577. doi:10.1002/dvdy.10317.

Mitsiadis TA, Pagella P, Cantù C. 2017. Early Determination of the Periodontal Domain by the Wnt-Antagonist Frzb/Sfrp3. Frontiers in Physiology. 8. [accessed 2022 Aug 16]. https://www.frontiersin.org/articles/10.3389/fphys.2017.00936.

Neves VCM, Babb R, Chandrasekaran D, Sharpe PT. 2017. Promotion of natural tooth repair by small molecule GSK3 antagonists. Sci Rep. 7(1):39654. doi:10.1038/srep39654.

Pirinen M, Lappalainen T, Zaitlen NA, GTEx Consortium, Dermitzakis ET, Donnelly P, McCarthy MI, Rivas MA. 2015. Assessing allele-specific expression across multiple tissues from RNA-seq read data. Bioinformatics. 31(15):2497–2504. doi:10.1093/bioinformatics/btv074.

Wang C, Wang Y, Wang H, Yang H, Cao Yangyang, Xia D, Cao Yu, Fan Z. 2021. SFRP2 enhances dental pulp stem cell-mediated dentin regeneration in rabbit jaw. Oral Diseases. 27(7):1738–1746. doi:10.1111/odi.13698.
